# Supplementary material for: Comparisons of retinal vessel density and glaucomatous parameters in optical coherence tomography angiography
Source: PLoS One. 2020 Jun 25;15(6):e0234816. doi: 10.1371/journal.pone.0234816 (PMC7316331; doi:10.1371/journal.pone.0234816)
Supplement: Attachment — Submitted filename: Response to Reviewers.docx [file pone.0234816.s003.docx]

Dear editor and reviewers:

It is with excitement that I resubmit to you a revised version of manuscript “Comparisons of retinal vessel density and glaucomatous parameters in optical coherence tomography angiography” for the "PLOS ONE ". Thank you for your guidance in revising my manuscript and giving me the opportunity to resubmit this manuscript. We are sending the revised manuscript according to the comments of the reviewers. Revised portion are rectified in red in “Revised Manuscript with Track Changes”. We have responded specifically to each suggestion below, beginning with your own.

Reviewer #3: The content of this manuscript is now sound and fine, though not particularly novel findings. There are a lot of formatting issues. For example, there are non-English characters present in the figures. And also, other font is used among Times New Roman in the main text, which looks messy. Systematic language and formatting editing are needed before being published. Lastly, age typically only needs one digit after the decimal point and correlation only needs two digits after the decimal.

Response:

Following the suggestion of reviewer, I have corrected non-English characters mistakes in Figure 1c and Figure 2b. Age has been changed to remain one digit after the decimal point in the main text (Line 158) and Table 1. Pearson’s correlation coefficients are usually remained three digits after the decimal point in a lot of references, so, I did not do any changes. But, in order to look more clarity, I did some formatting changes in Table 3.

Thanks for the help of reviewers and editor, I hope that the revised manuscript is now suitable for publication. Certainly, we are pleased to get more advice from you.

Best wish.

Li Zhen
